# Effects of a fixed low-dose ropivacaine with different volume and concentrations on interscalene brachial plexus block: a randomized controlled trial

**DOI:** 10.1186/s12871-016-0248-4

**Published:** 2016-09-30

**Authors:** Wenwen Zhai, Xuedong Wang, Yulan Rong, Min Li, Hong Wang

**Affiliations:** 1 Department of Anesthesiology, Peking University Third Hospital, No. 49, Hua Yuan North Street, Hai Dian District Beijing, China; 2 Department of Anesthesiology, West Virginia University, 6245 Inkster Rd., Garden City, MI 48135 USA

**Keywords:** Interscalene brachial plexus block, Shoulder arthroscopy, Sensory block, Motor block, Analgesia, Dose-response relationship

## Abstract

**Background:**

Ultrasound guidance has reduced the amount of local anesthetics to achieve a successful block. Previous studies of the relationship between the volume or concentration of local anesthetics and the effects of the block were based on relatively high doses of local anesthetics. We tested the hypothesis that providing low dose of ropivacaine at three combinations of volumes and concentrations for ultrasound-guided interscalene brachial plexus block would produce different effects in the aspect of onset time, pain control and the incidence of side effects.

**Methods:**

Ninety-nine patients undergoing elective arthroscopic shoulder surgery were randomized to receive an ultrasound guided combined with nerve stimulator mediated interscalene block with ropivacaine 0.75 % (6.7 ml, Group 0.75), 0.5 % (10 ml, Group 0.5) or 0.25 % (20 ml, Group 0.25). The primary end point was the onset time of the sensory blockade, assessed by using a pinprick in the C5-6 dermatome. The secondary end points included the onset time of the motor blockade, block success rate, postoperative pain rating score, rescue analgesics requirement, sleep quality, strength of the hand on the block side,and the incidence of hemi-diaphragmatic paresis which was evaluated by ultrasonography.

**Results:**

There was a statistically significant difference of the sensory block median onset times among Group 0.75 (5 min), Group 0.5 (10 min) and Group 0.25 (20 min). One patient in Group 0.5 and 20 patients in Group 0.25 did not achieve a complete motor block within 30 min, which were also significantly different. No significant difference was observed in postoperative analgesia, decrease of handgrip strength and the incidence of hemi-diaphragmatic paresis among the 3 groups.

**Conclusions:**

This study demonstrates that ropivacaine 50 mg as 0.25, 0.5 or 0.75 % solution for interscalene brachial plexus block before arthroscopic shoulder surgery produces comparable blockade with few side effects, while 0.75 % seems to be more preferable as it is associated with faster onset time.

**Trial registration:**

ChiCTR-TRC-13004058. Registered 4 December 2013.

## Background

Interscalene brachial plexus block (ISBPB) is one of the most reliable and the most commonly performed techniques to control intra and postoperative pain [1, 2]. Ultrasound guidance with or without nerve stimulator has reduced the amount of local anesthetics (LA) to achieve a successful block, which may minimize complications of ISBPB. Previous studies have suggested that ISBPB with 27 mg of 0.75 % or 25 mg of 0.5 or 0.25 % low-dose ropivacaine were effective to achieve sensory and motor block for arthroscopic shoulder surgery [3–5].

Although low dose ropivacaine with the concentration arranging from 0.25 to 0.75 % has been used in ISBPB [3–5], the optimal combination of concentration and volume of such low dose has not been studied. Previous studies have used a fixed large dose of LA in different volumes and concentrations for a sciatic nerve block [6, 7], infraclavicular block [8], axillary block [9, 10], and humeral canal block [11]. The results of these studies with the respects of onset time, successful rate and block duration were not consistent. Various factors, including the technique used, the anatomic aspects of the injection and the pharmacodynamics aspect of LA may influence the results.

The ideal concentration-volume ratio for low dose ISBPB needs further study. We tested the hypothesis that 50 mg ropivacaine of three combinations of volumes and concentrations for ultrasound-guided ISBPB produces different effects in the aspect of onset time, pain control and the incidence of side effects.

## Methods

Following institutional review board approval (Peking University Third Hospital Ethics Committee) and trial registration (ChiCTR-TRC-13004058), patients scheduled for elective arthroscopic shoulder surgery were recruited in this prospective, randomized, controlled, double blind clinical trial. Written informed consents were obtained from all patients.

The inclusion criteria were age between 18 and 80 years and an American Society of Anesthesiologists physical status of I and II. The exclusion criteria were patient refusal, neurologic or neuromuscular disease, severe bronchopulmonary disease, coagulation disorders, infection at the injection site, known allergy to one or more medications used in the study protocol, body mass index over 32 kg/m^2^ and patients treated with opioid analgesics.

Using a computer generated random number table, patients were randomized into three groups: 6.7 ml of 0.75 % ropivacaine (Group 0.75), 10 ml of 0.5 % ropivacaine (Group 0. 5) and 20 ml of 0.25 % ropivacaine (Group 0.25). Sealed opaque envelopes with the study group allocation were opened before the blocks were performed. An independent anesthesiologist who was not involved in the study prepared the study solution.

Routine monitors (pulse oximeter, noninvasive blood pressure cuff, and electrocardiogram) were used, and intravenous access was established. Patients were premedicated intravenously with midazolam 2 mg and dexamethasone 10 mg.

After skin disinfection and infiltration with lidocaine 1 %, the scalene muscles and interscalene brachial plexus were imaged in the short axis using a 38-mm 13–6 MHz linear array ultrasound probe (Turbo SonoSite HFL, Bothell, WA), the transverse process of vertebra C7 was identified by the absence of the anterior tubercle, subsequently, root C7 of the brachial plexus was visualized in short-axis view, then move the probe to detect root C6 and C5. After clear identification of root C6 and C5, then the area posterior to the plexus was analysed for the presence of the dorsal scapular and long thoracic nerves located within or around the middle scalene muscle as a discrete hyperechoic structure containing hypoechoic fascicles within them. Then a short bevel 100-mm, 19-gauge stimulating needle (StimuLong Sono Tsui set, Pajunk, Geisingen, Germany) connected to a Stimuplex (Stimuplex-HNS II A; B. Braun Melsungen AG, Germany) nerve stimulator was inserted using an in-plane technique from posterior to the probe, following a shallower needle trajectory to avoid entering the bulk of middle scalene muscle. If the dorsal scapular and long thoracic nerves had been visualized within the middle scalene, avoid injuring them during the needle passage. But if they were hard to identify by ultrasound, during inserting the stimulating needle, the contractions of the serratus anterior, rhomboids, and levator scapulae could help identify the dorsal scapular (rhomboids and levator scapulae muscle) or long thoracic (serratus anterior muscle), and once these muscle twitches were observed, stop and change the needle passage to avoid nerve injury. Elicitation of a sustained deltoid motor response at 0.3–0.5 mA with the tip of the needle positioned just lateral to the C5-6 roots confirmed the correct placement of the needle. All injections were administered slowly with repeated aspiration to prevent or detect early intravascular injection. The blocks were performed by an experienced physician who specialized in ultrasound and nerve stimulation–assisted interscalene block. After completion of the block, all the evaluations were performed by another blinded independent observer who was not involved in grouping, study solution preparing and interscalene block performing. All patients were blind to their group throughout the study.

Sensory and motor blockades were assessed every 5 min for up to 30 min. Sensory blockade was assessed by using a pinprick in the C5-6 dermatome (lateral region of the arm, innervated by the superior cutaneous nerve of the arm, branch of the axillary nerve) on a 3-point verbal rating scale: 0, normal sensation; 1, dull sensation (analgesia); and 2, no sensation (anesthesia). The onset time for a sensory block was defined as the time elapsed between the end of the block procedure and the moment when the pinprick test yielded a score of 2. Failure to reach a score of 2 within 30 min of interscalene block was considered to be block failure. Motor blockade was determined by loss of shoulder abduction (deltoid sign) and was objectively assessed on a 3-point rating scale: 0, normal movement; 1, diminished movement; and 2, no movement.

Complications, such as hematoma, Horner’s syndrome, hoarseness, respiratory distress and a decrease in SpO_2_ of more than 5 %, were also assessed during this period.

Before and 30 min after the block, an independent observer evaluated ipsilateral hemidiaphragmatic movement on deep inspiration by ultrasonography using a 5–2 MHz broadband curved array transducer (SonoSite, Inc., Bothell, Wash). Patients were examined in the upright sitting position and scanned from a low intercostal or subcostal approach using the liver or spleen as an acoustic window [3]. Normal caudad movement with deep inspiration was considered to be no paresis, whereas no movement or paradoxical cephalad movement was designated as paresis.

General anesthesia was induced with propofol (target controlled infusion 3–5 μg/ml), fentanyl (1.5 ~ 2.5 μg/kg) and cisatracurium (0.15 mg/kg) and maintained with propofol (target controlled infusion 3–5 μg/ml) and remifentanil (continuous infusion 0.05–0.3 μg/kg/min). Patients were intubated and ventilated with an oxygen/air admixture. Anesthesia management was left to the discretion of the attending anesthesiologist. No other analgesics were administered intraoperatively.

All patients received parecoxib 40 mg bid for the first two days after surgery. Tramadol 100 mg or pethidine 50 mg was given as rescue analgesics for numerical rating pain scale (NRPS) ≥4 or by the patient’s request.

Patients were instructed to rate their pain using an 11-point NRPS ranging from 0 to 10 (0, no pain; 10, worst imaginable pain). NRPS of rest pain was measured before the block, right before discharge from the PACU and at 4, 8, 24 h after the block. The timing and dosage of analgesics were recorded. Twenty-four hours after the block, patients were questioned for NRPS of the worst pain, time of the first shoulder pain, sleep quality (0 = no sleep disturbance because of pain, 1 = sleep disturbance because of pain) and satisfaction with the interscalane block (0–10, 0 = very unsatisfied; 10 = very satisfied).

The blinded investigator recorded grip strength in the operative limb using the same Jamar dynamometer before the administration of intravenous sedation, right before discharge from the PACU and at 4, 8 and 24 h after the block.

The patient was telephone interviewed if they suffered a late complication such as scapula alata, nerve injury and pain along the medial border of scapula or radiating to the arm and forearm related to interscalene block after more than one year since discharge from hospital.

The primary outcome was onset time. Our research hypothesis was that compared with low concentration/high volume group, high concentration/low volume group would result in a shorter onset time. According to our preliminary test, the onset time for 10 mL of 0.5 % ropivacaine is 10 ± 5 min. We considered a time difference of 50 % as being clinically relevant. To obtain a 2-tailed α error of 0.05 and a statistical power of 0.9, the calculated sample size was 27 patients per group. We recruited 33 patients per group in anticipation of 20 % potential dropouts.

### Statistical analysis

The SPSS version 20.0 (SPSS, Inc., Chicago, USA) was used. The normality of the continuous data was first assessed with the Kolmogorov-Smirnov test. Normally distributed variables were expressed as the mean ± SD and compared between groups using one way analysis of variance (ANOVA) with an LSD post hoc test. Non-normally distributed variables were expressed as the median (range) and analysed using the Kruskal-Wallis H test. Categorical variables were expressed as the number (%) and compared between groups using a Pearson test. Kaplan-Meier survival curves were constructed (survival was equivalent to unsuccessful block). The log-rank test was used to detect differences in block onset times among groups. *P* < 0.05 was considered statistically significant.

## Results

**Fig. 1:**
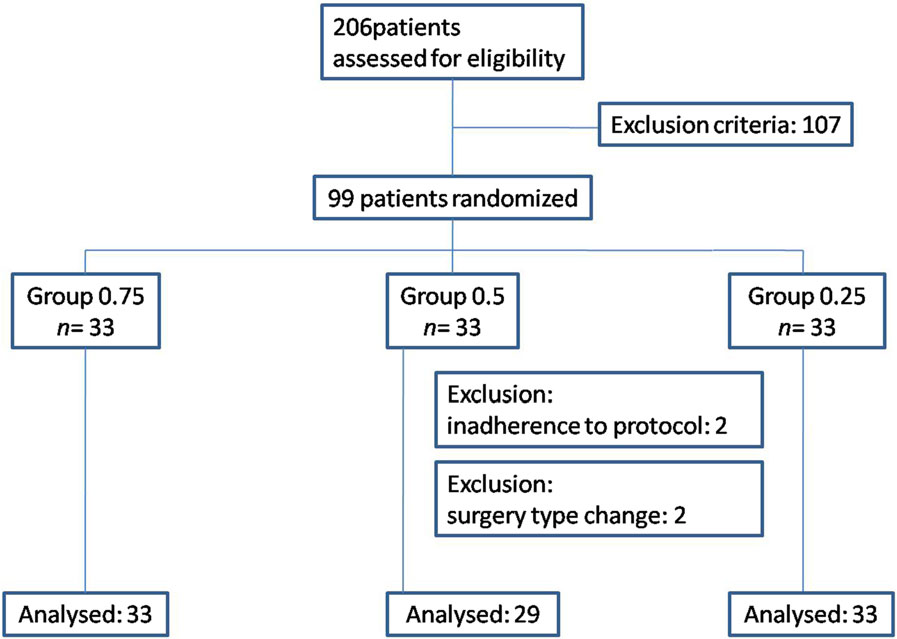
Allocation process according to CONSORT

From December 2013 to February 2014, 99 patients were enrolled in the study. Four patients were excluded either due to the variation from the protocol or the changes of the procedure. Figure 1 presented the allocation process according to the Consolidated Standards of Reporting Trials (CONSORT) statement. No differences in patient demographic data were observed among the 3 groups (Table 1).

**Table 1:** Patient demographic data

	Group 0.75 (*n* = 33)	Group 0.5 (*n* = 29)	Group 0.25 (*n* = 33)	*P*
Age, yr	45 ± 13	44 ± 16	49 ± 16	0.416
Height, cm	165 ± 9	169 ± 11	168 ± 7	0.211
Weight, kg	67 ± 13	77 ± 13	71 ± 11	0.219
BMI, kg/m^2^	24.3 ± 3.5	24.8 ± 2.6	25.3 ± 3.3	0.478
Sex (male/female), n	15/18	17/12	24/9	0.079
Surgical type (Bankart repair/Rotator cuff repair), n	8/25	7/22	7/26	0.655
Surgical time, min	101 ± 40	87 ± 32	103 ± 40	0.197
Remifentanil consumption, μg/kg/min	0.07 ± 0.03	0.08 ± 0.03	0.07 ± 0.03	0.619

Continuous variables are presented as the means ± SDs; categorical variables are presented as counts

**Fig. 2:**
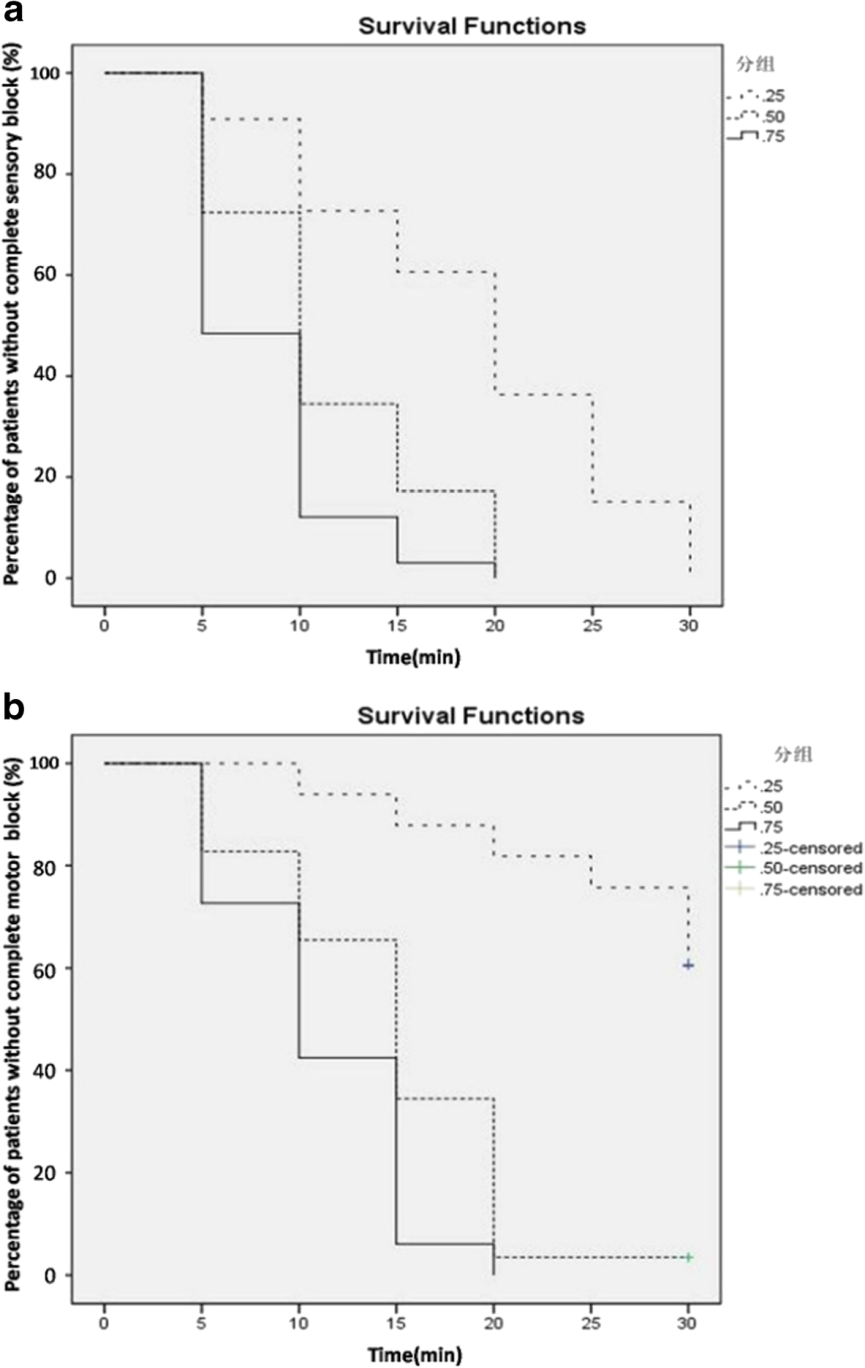
Kaplan-Meier survival curves constructed using survival as being equivalent to an “unsuccessful block.” The onset of sensory (**a**) and motor (**b**) blockade was shorter in patients receiving higher concentration of ropivacaine (*P* = 0.000 by log-rank test)

The onset of sensory and motor blockade was shorter in patients receiving higher concentrations of ropivacaine (*P* < 0.05, Fig. 2). All patients received full sensory block when the 30-min follow-up ended. The median (range) sensory blockade onset times, which were 5 (5–20) minutes, 10 (5–20) minutes, and 20 (5–30) minutes for Group 0.75, 0.5 and 0.25 respectively. They were statistically significantly different (*P* = 0.000 by log-rank test). Group 0.75 had faster onset time than group 0.5 (*P* = 0.013 by log-rank test). The median (range) of motor blockade onset times of Group 0.75 and Group 0.5 were 10 (5–25) minutes and 15 (5–30) minutes, respectively. One patient from Group 0.5 and 20 patients from Group 0.25 did not achieve complete motor blockade when the 30-min follow-up ended. These differences were also significantly different (*P* = 0.000 by log-rank test) among the three groups. There was no significant difference between group 0.75 and group 0.5 after further comparison.

Baseline diaphragmatic movements were similar and normal in all patients. Thirty minutes after ISBPB, absent or paradoxical diaphragmatic movements were observed in 58 % of Group 0.75, 69 % of Group 0.5, and 70 % of Group 0.25 (*P* = 0.516), respectively.

In Group 0.25, one patient developed Horner’s syndrome 30 min after ISBPB. No patients suffered from respiratory distress, hypoxia, post-block hoarseness, hematoma or LA toxicity.

The pain scores (NRPS) measured before the block, right before discharge from the PACU, and 4, 8, 24 h after block and the worst pain were similar in all groups. The percentages of the worst pain scores over 4, rescue analgesia, sleep quality, time to first report of pain and satisfaction scores were also comparable among the 0.75, 0.5, and 0.25 groups. (Table 2)

**Table 2:** Outcomes of pain control among the 3 groups

	Group 0.75	Group 0.5	Group 0.25	*P*
NRPS in the PACU	0 (0–2)	0 (0–1)	0 (0–0)	0.581
NRPS 4 h after ISB	0 (0–2)	0 (0–1)	0 (0–3)	0.771
NRPS 8 h after ISB	0 (0–3)	0 (0–3)	0 (0–3)	0.211
NRPS 24 h after ISB	1 (0–6)	1 (0–5)	0 (0–7)	0.439
Worst pain score	3 (0–6)	3 (0–7)	3 (0–7)	0.882
Percentage of worst pain score over 4 (n,%)	11/33 (33 %)	6/29 (21 %)	12/33 (36 %)	0.372
Sleep disturbance because of pain (n,%)	6/33 (18 %)	2/29 (7 %)	5/33 (15 %)	0.416
Satisfaction score	9.2 ± 1.3	8.9 ± 1.2	9.4 ± 1.0	0.167

Data are the mean ± SD, median (range) or numbers (incidence)

**Fig. 3:**
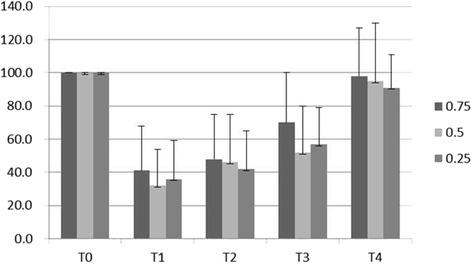
Summary of the averages of the hand strength changes in patients receiving 0.75 %, 0.5 % and 0.25 % ropivacaine. Measurements were made preoperatively (T0), in the PACU (T1), and 4 h (T2), 8 h (T3) and 24 h (T4) after the interscalene block. The values represent the mean ± sd

The preoperative values of hand grip strength were used as 100 %, and the average of quantitative preoperative grip strength was 24.0 ± 11.5 kg, 27.6 ± 12.9 kg and 26.6 ± 9.6 kg for Group 0.75, Group 0.5 or Group 0.25, respectively. The recovery time of the hand grip strength of the blocked arm was similar among the three groups during the study period. The strength of the hand on the blocked side decreased to 41, 32 and 36 % in the PACU. At 4 h after the block, the strength recovery was 48, 46 and 42 % in Group 0.75, Group 0.5 or Group 0.25, respectively. At 8 h after the block, the hand strength was 70, 52 and 57 % in Group 0.75, Group 0.5 or Group 0.25, respectively. At 24 h after the block, the hand grip strength restored to 98, 95 and 91 %. (Fig. 3)

Over 85 % of the patients completed the telephone interview and none reported a late and severe complication related to interscalene block such as scapula alata.

## Discussion

This prospective, randomized, double blind trial compared the clinical effects of 3 combinations of volumes and concentrations of ropivacaine (6.7 ml of 0.75 %, 10 ml of 0.5 % and 20 ml of 0.25 %) for ISBPB. The results demonstrated a faster onset of complete sensory and motor blockade for the higher concentration groups. No intergroup differences were found in terms of the block success rate, postoperative analgesia, decrease in hand grip strength and incidence of hemi-diaphragmatic paresis. Interscalene block is one of the most reliable and most commonly performed regional techniques for arthroscopic shoulder surgery. Clinicians prefer to choose the regimen with fast onset, high success rate, reliable analgesic effect, early recovery of hand motor and good preservation of diaphragm function. According to our results, except onset time, there was no significant difference among the three groups in these aspects. To facilitate operation and shorten OR turnover time, ropivacaine 0.75 % seems to be more preferable.

Both concentration and volume of LA were crucial factors for the onset of block. In theory, an increased LA concentration can shorten the onset time because of the improved neural penetration by LA molecules [12], and an increased volume can shorten the onset time by promoting LA diffusion around neural structures [10]. However, how the volume/concentration ratio in a fixed dose affects the regional anesthetic block remains unclear. The results of previous studies on the onset time of the same dose of LA diluted in different volumes for nerve blocks [6–11] were inconsistent, which might be attributed to volume/concentration ratio and the following factors as well.

First, the type of the tissue surrounding the target nerve is essential for the onset of a nerve block. It has been suggested that the LA volume was the main determinant of the onset time for a single-injection axillary block [10], where only loose connective tissue surrounded the brachial plexus. For Labat sciatic nerve block, where the sciatic nerve was surrounded by compact structures with minimal compliant, such as bone and muscles, a higher concentration seemed to be more effective [7].

Second, multiple injection technique may eliminate volume/concentration ratio effect. For example, using a single injection technique, with a fixed dose of LA diluted to different volumes, volume/concentration ratio affected the onset of axillary block [10]. But using a multiple injection technique, the onsets of an axillary block were similar among different volume/concentration ratio groups [9]. A study of single injection Labat sciatic nerve block showed various volume/concentration combinations resulted in different onset times [7]. While using a double injection technique, the onset of Labat sciatic nerve block did not differ with the different volume/concentration ratio [6]. Multiple injection facilitated the injection of LA targeting at each component of the peripheral nerve and resulted in a better distribution of LA solution around each individual nerve, with this distribution of LA, when proper doses were used, the anatomic characters became secondary to the injection technique and the effect of concentration/volume ratio was weakened. Study with a multiple injection technique in other area, such as humeral canal block [11], also showed no difference with different volume/concentration combinations.

Analgesia for shoulder surgery requires blockade of the C5-6 nerve roots or superior trunk, which is divided into suprascapular, axillary and lateral pectoral nerves innervating the shoulder [2]. Therefore, we used a single injection technique targeting only the C5-6 roots with the guidance of ultrasound and a nerve stimulator. We found that increasing the ropivacaine concentration from 0.25 to 0.5 or 0.75 % resulted in a faster onset of both sensory and motor blocks. At the level of the interscalene groove, the scalene muscles and their investing fascia provided a well-demarcated potential space in which LA could be injected [13]. The anatomic aspects of tissue surrounding the C5-6 roots might explain why a higher concentration led to faster onset.

Previous studies have shown that 50 mg of low dose ropivacaine diluted into 0.75 % of 6.7 ml [3], 0.5 % of 10 ml [4] and 0.25 % of 20 ml [5] were proved to be effective in different studies for at least two hours after the shoulder surgery. In our study, all three groups had satisfactory shoulder analgesia for at least 8 h after the interscalane nerve block with 50 mg of ropivacaine. Between 8 and 24 h after the block, 30 % of patients experienced their worst pain score over 4, and almost all of these patients were confirmed to have massive rotator cuff tear. It suggested that for surgeries of massive rotator cuff tear, better pain control including a relatively large dose of LA, multimodal analgesia or a continuous interscalene block should be selected according to the laceration degree of the rotator cuff.

Our study was the first to compare the incidence of hemidiaphragmatic paresis with same dose of LA diluted in different volumes. The phrenic nerve arises mainly from the C4 root, with variable contributions from C3 and C5. It courses caudally between the ventral surface of the anterior scalene muscle and prevertebral fascial layer that covers this muscle and is therefore separated from the brachial plexus only by a thin fascial layer. As a result, its block in ISBPB can be explained by the proximity to the brachial plexus or to the cephalad spread of local anesthetic to the C3–5 roots of the cervical plexus before their formation of the phrenic nerve [4]. On the basis of the anatomic characteristics, it had been suggested that reducing the dose of LA [4, 14–16] or moving the injection site farther from the C3-5 roots [17] may be able to lower the incidence. In our study, with the same LA dosage and targeting nerve, hemidiaphragmatic paresis occurred in 58-70 % of patients 30 mins after block, with no statistical difference among the three groups, and the incidence was similar to those reported in previous studies [4].

Our study has some limitations. First, the blockade was performed by an anesthesiologist experienced with interscalane block; therefore, our findings should be carefully applied to other conditions as this was a low dose study with as little as 6.7 ml of LA in one of the groups. Second, the result obtained from ropivacaine might not be true for other LAs. Third, compared to European population, the presented (low) BMI is conspicuous and relevant as for blockade effect with very low volumes of local anesthetics. Therefore, results cannot thoroughly be transferred. Forth, although the ratio of male and female patients was not significantly different among groups, there is an imbalance in the distribution of the gender of patients, so that an influence of gender may not be excluded. At last, although a common approach to perform ultrasound-guided interscalene block is to image the brachial plexus in short axis and to insert the needle using the in-plane approach [3, 4, 14, 16–18], this technique bears the risk of injuring the small branches of the brachial plexus associated with the middle scalene muscle, such as the dorsal scapular and long thoracic nerves [19, 20]. Unfortunately, neither outcome data describing injury to these nerves, nor technical guideline to ensure avoidance of this event is available. It is safer to follow a shallower needle trajectory to avoid entering the bulk of middle scalene muscle, visualize these nerves by ultrasound, and use a peripheral nerve stimulator to identify proximity to these nerves. To conclude, extra caution should be taken using short-axis in-plane brachial plexus block to avoid the nerve damage.

## Conclusion

In summary, the present study shows that ropivacaine 50 mg given around interscalene brachial plexus as 0.25, 0.5 or 0.75 % solution before arthroscopic shoulder surgery produces comparable blockade with few side effects, while 0.75 % seems to be more preferable as it is associated with faster onset time.

## Abbreviations

ANOVA: Analysis of variance
CONSORT: Consolidated standards of reporting trials
ISBPB: Interscalene brachial plexus block
LA: Local anesthetics
NRPS: Numerical rating pain scale
PACU: Post-anesthctic care unit
SpO_2_: Pulse oxygen saturation
